# 4-Amino-8-cyclo­pent­yloxy-7-meth­oxy-2*H*-chromen-2-one monohydrate

**DOI:** 10.1107/S160053681201450X

**Published:** 2012-04-18

**Authors:** Man-Hua Ding, Xiao-Ping Jiang

**Affiliations:** a Department of Biology and Chemistry, Hunan University of Science and Engineering, Yongzhou Hunan 425100, People’s Republic of China

## Abstract

The asymmetric unit of the title compound, C_15_H_17_NO_4_·H_2_O, contains two organic mol­ecules with marginal differences between them and two water molecules. The chromine rings in both mol­ecules are essentially planar, with maximum deviations of 0.012 (2) and 0.060 (2) Å. The five-membered cyclo­pentane rings adopt envelope conformations in both mol­ecules. In the crystal, the components are linked by N—H⋯O, O—H⋯O and C—H⋯O hydrogen bonds, resulting in a three-dimensional network.

## Related literature

For applications of the title compound in the treatment or prevention of disease, see: Scherlach *et al.* (2011 ▶); Luan *et al.* (2011 ▶); Yang *et al.* (2011 ▶). For a related structure, see: Doriguetto *et al.* (2006 ▶). 
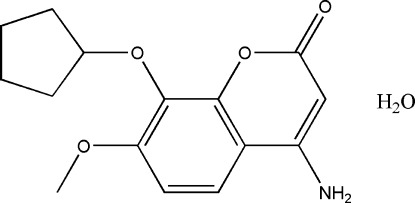


## Experimental

### 

#### Crystal data

C_15_H_17_NO_4_·H_2_O
*M* *_r_* = 293.31
Monoclinic, 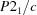

*a* = 20.3651 (4) Å
*b* = 7.43162 (16) Å
*c* = 19.4049 (4) Å
β = 91.0792 (18)°
*V* = 2936.32 (11) Å^3^
*Z* = 8
Cu *K*α radiation
μ = 0.83 mm^−1^
*T* = 153 K
0.36 × 0.31 × 0.20 mm

#### Data collection

Agilent Xcalibur Atlas Gemini ultra diffractometer
Absorption correction: multi-scan (*CrysAlis PRO*; Agilent, 2006 ▶) *T*_min_ = 0.754, *T*_max_ = 0.851
13804 measured reflections
5167 independent reflections
4701 reflections with *I* > 2σ(*I*)
*R*_int_ = 0.026

#### Refinement

*R*[*F* ^2^ > 2σ(*F* ^2^)] = 0.054
*wR*(*F* ^2^) = 0.193
*S* = 1.08
5167 reflections
394 parameters
6 restraints
H-atom parameters constrained
Δρ_max_ = 0.38 e Å^−3^
Δρ_min_ = −0.30 e Å^−3^

### 

Data collection: *CrysAlis PRO* (Agilent, 2006 ▶); cell refinement: *CrysAlis PRO*; data reduction: *CrysAlis PRO*; program(s) used to solve structure: *SHELXS97* (Sheldrick, 2008 ▶); program(s) used to refine structure: *SHELXL97* (Sheldrick, 2008 ▶); molecular graphics: *SHELXTL* (Sheldrick, 2008 ▶); software used to prepare material for publication: *SHELXTL*.

## Supplementary Material

Crystal structure: contains datablock(s) I, global. DOI: 10.1107/S160053681201450X/pv2524sup1.cif

Structure factors: contains datablock(s) I. DOI: 10.1107/S160053681201450X/pv2524Isup2.hkl

Supplementary material file. DOI: 10.1107/S160053681201450X/pv2524Isup3.cml

Additional supplementary materials:  crystallographic information; 3D view; checkCIF report

## Figures and Tables

**Table 1:** Hydrogen-bond geometry (Å, °)

*D*—H⋯*A*	*D*—H	H⋯*A*	*D*⋯*A*	*D*—H⋯*A*
N1—H1*A*⋯O5^i^	0.88	1.99	2.861 (3)	172
N1—H1*B*⋯O2*W*^ii^	0.88	2.00	2.816 (3)	153
N2—H2*A*⋯O1^iii^	0.88	2.03	2.888 (3)	166
N2—H2*B*⋯O1*W*^i^	0.88	2.08	2.838 (3)	144
O1*W*—H1*AA*⋯O5^iv^	0.84	1.94	2.769 (3)	170
O2*W*—H2*AB*⋯O1^v^	0.84	1.94	2.767 (3)	168
O1*W*—H1*AB*⋯O7	0.84	2.17	2.956 (3)	156
O1*W*—H1*AB*⋯O8	0.84	2.39	2.994 (3)	130
O2*W*—H2*AA*⋯O3	0.84	2.00	2.833 (3)	171
C7—H7⋯O2*W*^ii^	0.95	2.60	3.493 (3)	158
C15—H15*B*⋯O2*W*^vi^	0.99	2.56	3.309 (4)	133

Symmetry codes: (i) 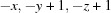; (ii) 
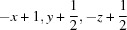
; (iii) 
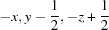
; (iv) 
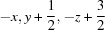
; (v) 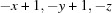; (vi) 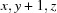.

**Fig. 1.**
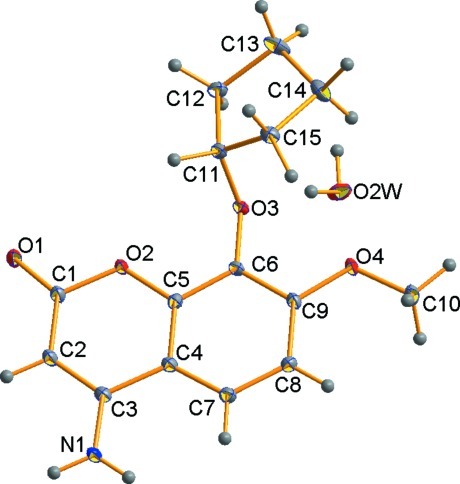
The molecular structure of the title compound (molecule I) with the atom numbering scheme. Displacement ellipsoids are drawn at the 30% probability level. H atoms are presented as small spheres of arbitrary radius.

**Fig. 2.**
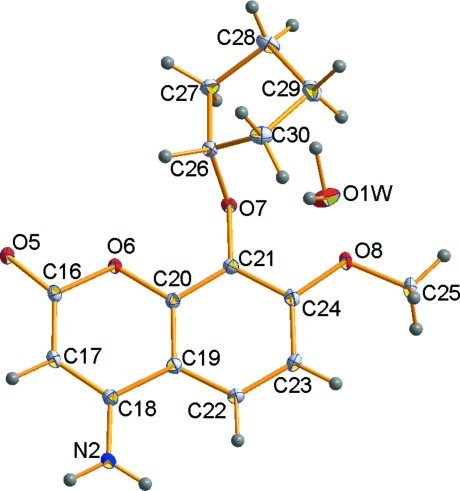
The molecular structure of the title compound (molecule II) with the atom numbering scheme. Displacement ellipsoids are drawn at the 30% probability level. H atoms are presented as small spheres of arbitrary radius.

**Fig. 3.**
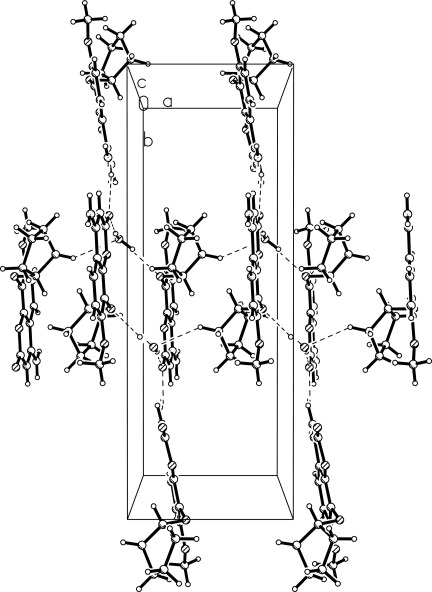
A view of the intermolecular hydrogen bonds (dotted lines) in the crystal structure of the title compound. H atoms non-participating in hydrogen-bonding were omitted for clarity.

## supplementary crystallographic information

### Comment

The title compound belongs to a class of important medicinal intermediates which may be useful for the treatment or prevention of inflammatory and other diseases (Scherlach *et al.*, 2011; Luan *et al.*, 2011; Yang *et al.*, 2011).

There are two crystallographically independent molecules of the title compound in an asymmetric unit labeled as molecule I (Fig. 1) and molecule II (Fig. 2) containing chromine rings (O2/C1–C9) and (O6/C16–C24), respectively. There are only marginal differences between the two molecules. The chromine rings (O2/C1–C9) and (O6/C16–C24) in both molecules are essentially planar with maximum deviations for atoms C9 and C16 being 0.012 (2) and 0.060 (2) Å, respectively. The five-membered cyclopentane rings adopt C14- and C29- envelope conformations with these atoms lying 0.581 (5) and 0.604 (5) Å, respectively, out of the planes formed by the remaining reings atoms. In the crystal, a three-dimensional network is established through the N—H···O, O—H···O and C—H···O hydrogen bonds which stabilizes the crystal structure (Fig. 3, Tab. 1).

### Experimental

To a solution of 8-(cyclopentyloxy)-4-hydroxy-7-methoxy -2*H*-chromen-2-one (0.292 g) in toluene (10 ml) were added ammonium acetate (1.63 g) and acetic acid (1.26 g). The resulting mixture was stirred at reflux for 2 h. The water formed was removed azeotropically using a Dean-Stark apparatus, then toluene was also removed. The resulting solution was stirred for an additional 3 h at reflux. The reaction mixture was quenched with ice water (20 ml). A filtration was performed. The filter cake was washed three times with H_2_O (20 ml). The solid was dried in an oven under reduced pressure and the product was recrystallized from ethyl acetate to afford yellow crystals of the title compound.

### Refinement

H atoms bonded to N and O atoms were located from a difference Fourier map and included in the refinement with distance restraints of O—H = 0.84 and N—H = 0.88 Å, and with *U*_iso_(H) = 1.2*U*_eq_(N) or 1.5*U*_eq_(O). Other H atoms were positioned geometrically and refined using a riding model, with C—H = 0.95–1.00 Å and with *U*_iso_(H) = 1.2 (1.5 for methyl groups) times *U*_eq_(C).

### Crystal data

**Table:**

C_15_H_17_NO_4_·H_2_O	*F*(000) = 1248
*M**_r_* = 293.31	*D*_x_ = 1.327 Mg m^−^^3^
Monoclinic, *P*2_1_/*c*	Cu *K*α radiation, λ = 1.54184 Å
Hall symbol: -P 2ybc	Cell parameters from 13804 reflections
*a* = 20.3651 (4) Å	θ = 4.6–67.0°
*b* = 7.43162 (16) Å	µ = 0.83 mm^−^^1^
*c* = 19.4049 (4) Å	*T* = 153 K
β = 91.0792 (18)°	Block, yellow
*V* = 2936.32 (11) Å^3^	0.36 × 0.31 × 0.20 mm
*Z* = 8	

### Data collection

**Table:**

Agilent Xcalibur Atlas Gemini ultra diffractometer	5167 independent reflections
Radiation source: fine-focus sealed tube	4701 reflections with *I* > 2σ(*I*)
Graphite monochromator	*R*_int_ = 0.026
ω scans	θ_max_ = 67.0°, θ_min_ = 4.6°
Absorption correction: multi-scan (*CrysAlis PRO*; Agilent, 2006)	*h* = −21→24
*T*_min_ = 0.754, *T*_max_ = 0.851	*k* = −8→8
13804 measured reflections	*l* = −21→23

### Refinement

**Table:**

Refinement on *F*^2^	Secondary atom site location: difference Fourier map
Least-squares matrix: full	Hydrogen site location: inferred from neighbouring sites
*R*[*F*^2^ > 2σ(*F*^2^)] = 0.054	H-atom parameters constrained
*wR*(*F*^2^) = 0.193	*w* = 1/[σ^2^(*F*_o_^2^) + (0.103*P*)^2^ + 4.5099*P*] where *P* = (*F*_o_^2^ + 2*F*_c_^2^)/3
*S* = 1.08	(Δ/σ)_max_ < 0.001
5167 reflections	Δρ_max_ = 0.38 e Å^−^^3^
394 parameters	Δρ_min_ = −0.30 e Å^−^^3^
6 restraints	Extinction correction: *SHELXL97* (Sheldrick, 2008), Fc^*^=kFc[1+0.001xFc^2^λ^3^/sin(2θ)]^-1/4^
Primary atom site location: structure-invariant direct methods	Extinction coefficient: 0.0031 (5)

### Special details

**Table:**

Experimental. Absorption correction: empirical absorption correction using spherical harmonics implemented in SCALE3 ABSPACK scaling algorithm (Agilent, 2006)
Geometry. All esds (except the esd in the dihedral angle between two l.s. planes) are estimated using the full covariance matrix. The cell esds are taken into account individually in the estimation of esds in distances, angles and torsion angles; correlations between esds in cell parameters are only used when they are defined by crystal symmetry. An approximate (isotropic) treatment of cell esds is used for estimating esds involving l.s. planes.
Refinement. Refinement of *F*^2^ against ALL reflections. The weighted *R*-factor *wR* and goodness of fit *S* are based on *F*^2^, conventional *R*-factors *R* are based on *F*, with *F* set to zero for negative *F*^2^. The threshold expression of *F*^2^ > σ(*F*^2^) is used only for calculating *R*-factors(gt) *etc*. and is not relevant to the choice of reflections for refinement. *R*-factors based on *F*^2^ are statistically about twice as large as those based on *F*, and *R*- factors based on ALL data will be even larger.

### Fractional atomic coordinates and isotropic or equivalent isotropic displacement parameters (Å^2^)

**Table:**

	*x*	*y*	*z*	*U*_iso_*/*U*_eq_	
O1	0.31349 (9)	0.6751 (3)	0.05051 (9)	0.0277 (5)	
O2	0.40956 (8)	0.6823 (3)	0.10419 (9)	0.0196 (4)	
O3	0.53980 (8)	0.6560 (2)	0.08977 (8)	0.0189 (4)	
O4	0.62167 (8)	0.6828 (3)	0.19846 (9)	0.0251 (5)	
N1	0.32021 (10)	0.7551 (3)	0.29237 (11)	0.0239 (5)	
H1A	0.2771	0.7601	0.2944	0.029*	
H1B	0.3443	0.7650	0.3303	0.029*	
C1	0.34144 (12)	0.6908 (4)	0.10708 (13)	0.0214 (6)	
C2	0.31268 (12)	0.7164 (4)	0.17122 (14)	0.0227 (6)	
H2	0.2662	0.7230	0.1732	0.027*	
C3	0.34885 (12)	0.7326 (4)	0.23200 (13)	0.0194 (5)	
C4	0.42038 (12)	0.7240 (3)	0.22795 (13)	0.0183 (5)	
C5	0.44727 (12)	0.6996 (3)	0.16325 (13)	0.0174 (5)	
C6	0.51502 (12)	0.6893 (3)	0.15391 (12)	0.0180 (5)	
C7	0.46357 (12)	0.7384 (4)	0.28460 (13)	0.0203 (6)	
H7	0.4464	0.7561	0.3293	0.024*	
C8	0.53058 (13)	0.7274 (4)	0.27689 (13)	0.0212 (6)	
H8	0.5590	0.7377	0.3160	0.025*	
C9	0.55674 (12)	0.7012 (3)	0.21161 (13)	0.0195 (5)	
C10	0.66766 (13)	0.7058 (4)	0.25445 (14)	0.0281 (6)	
H10A	0.7124	0.6893	0.2378	0.042*	
H10B	0.6589	0.6166	0.2903	0.042*	
H10C	0.6632	0.8271	0.2736	0.042*	
C11	0.54069 (13)	0.8129 (4)	0.04407 (13)	0.0215 (6)	
H11	0.4969	0.8737	0.0423	0.026*	
C12	0.55964 (14)	0.7436 (4)	−0.02734 (13)	0.0262 (6)	
H12A	0.5295	0.7927	−0.0633	0.031*	
H12B	0.5575	0.6106	−0.0289	0.031*	
C13	0.63040 (16)	0.8087 (5)	−0.03896 (17)	0.0371 (8)	
H13A	0.6577	0.7101	−0.0573	0.044*	
H13B	0.6308	0.9105	−0.0718	0.044*	
C14	0.65589 (14)	0.8678 (4)	0.03183 (16)	0.0317 (7)	
H14A	0.6904	0.9609	0.0278	0.038*	
H14B	0.6738	0.7645	0.0583	0.038*	
C15	0.59466 (12)	0.9438 (4)	0.06545 (14)	0.0246 (6)	
H15A	0.6002	0.9477	0.1162	0.030*	
H15B	0.5849	1.0665	0.0483	0.030*	
O5	−0.18130 (8)	0.1918 (3)	0.70123 (9)	0.0248 (5)	
O6	−0.08754 (8)	0.2503 (3)	0.65200 (9)	0.0200 (4)	
O7	0.04038 (8)	0.3412 (2)	0.67260 (9)	0.0199 (4)	
O8	0.12364 (8)	0.3338 (3)	0.56941 (9)	0.0251 (5)	
N2	−0.17177 (11)	0.1820 (3)	0.45783 (11)	0.0255 (5)	
H2A	−0.2141	0.1598	0.4532	0.031*	
H2B	−0.1475	0.1940	0.4211	0.031*	
C16	−0.15351 (12)	0.2038 (4)	0.64569 (13)	0.0201 (6)	
C17	−0.18055 (12)	0.1794 (4)	0.57953 (13)	0.0217 (6)	
H17	−0.2258	0.1492	0.5751	0.026*	
C18	−0.14447 (12)	0.1972 (4)	0.52039 (13)	0.0200 (6)	
C19	−0.07400 (12)	0.2311 (4)	0.52828 (13)	0.0193 (5)	
C20	−0.04892 (12)	0.2580 (4)	0.59489 (13)	0.0183 (5)	
C21	0.01742 (12)	0.2956 (4)	0.60801 (13)	0.0189 (5)	
C22	−0.03026 (13)	0.2365 (4)	0.47378 (13)	0.0219 (6)	
H22	−0.0462	0.2182	0.4280	0.026*	
C23	0.03603 (13)	0.2680 (4)	0.48542 (13)	0.0224 (6)	
H23	0.0653	0.2691	0.4478	0.027*	
C24	0.05997 (12)	0.2983 (4)	0.55240 (14)	0.0207 (6)	
C25	0.17107 (13)	0.3265 (4)	0.51606 (15)	0.0283 (6)	
H25A	0.2149	0.3496	0.5358	0.043*	
H25B	0.1606	0.4178	0.4811	0.043*	
H25C	0.1702	0.2069	0.4947	0.043*	
C26	0.04488 (13)	0.1944 (4)	0.72309 (13)	0.0241 (6)	
H26	0.0012	0.1355	0.7292	0.029*	
C27	0.06993 (14)	0.2795 (5)	0.79080 (14)	0.0333 (7)	
H27A	0.0707	0.4123	0.7868	0.040*	
H27B	0.0411	0.2460	0.8293	0.040*	
C28	0.13955 (14)	0.2063 (4)	0.80336 (16)	0.0319 (7)	
H28A	0.1695	0.3028	0.8198	0.038*	
H28B	0.1395	0.1083	0.8379	0.038*	
C29	0.16034 (14)	0.1360 (4)	0.73356 (16)	0.0325 (7)	
H29A	0.1945	0.0420	0.7385	0.039*	
H29B	0.1770	0.2344	0.7043	0.039*	
C30	0.09641 (14)	0.0582 (4)	0.70385 (14)	0.0291 (6)	
H30A	0.0987	0.0450	0.6532	0.035*	
H30B	0.0869	−0.0607	0.7244	0.035*	
O1W	0.13246 (12)	0.6480 (3)	0.66569 (10)	0.0392 (6)	
H1AA	0.1427	0.6601	0.7076	0.059*	
H1AB	0.1154	0.5456	0.6612	0.059*	
O2W	0.63303 (11)	0.3753 (3)	0.07811 (10)	0.0325 (5)	
H2AA	0.6077	0.4646	0.0784	0.049*	
H2AB	0.6437	0.3589	0.0370	0.049*	

### Atomic displacement parameters (Å^2^)

**Table:**

	*U*^11^	*U*^22^	*U*^33^	*U*^12^	*U*^13^	*U*^23^
O1	0.0165 (9)	0.0481 (13)	0.0185 (9)	0.0032 (8)	−0.0009 (7)	−0.0048 (8)
O2	0.0114 (8)	0.0306 (10)	0.0168 (9)	0.0015 (7)	0.0012 (6)	−0.0020 (7)
O3	0.0168 (8)	0.0235 (10)	0.0165 (9)	0.0023 (7)	0.0044 (6)	0.0003 (7)
O4	0.0110 (8)	0.0421 (12)	0.0222 (10)	0.0022 (8)	0.0008 (7)	−0.0008 (8)
N1	0.0139 (10)	0.0391 (14)	0.0188 (11)	−0.0010 (9)	0.0034 (8)	−0.0013 (10)
C1	0.0152 (12)	0.0268 (14)	0.0222 (13)	0.0013 (10)	0.0007 (10)	−0.0015 (10)
C2	0.0139 (12)	0.0306 (15)	0.0237 (13)	0.0003 (10)	0.0034 (10)	−0.0012 (11)
C3	0.0176 (12)	0.0194 (13)	0.0214 (13)	0.0000 (10)	0.0037 (10)	−0.0003 (10)
C4	0.0169 (12)	0.0179 (13)	0.0201 (13)	0.0005 (9)	0.0028 (10)	0.0004 (10)
C5	0.0180 (12)	0.0168 (12)	0.0173 (12)	0.0001 (9)	−0.0003 (9)	0.0003 (9)
C6	0.0167 (12)	0.0207 (13)	0.0168 (12)	0.0016 (10)	0.0034 (9)	0.0000 (10)
C7	0.0202 (13)	0.0224 (14)	0.0184 (12)	0.0010 (10)	0.0031 (10)	−0.0001 (10)
C8	0.0190 (12)	0.0273 (14)	0.0173 (13)	0.0003 (10)	−0.0005 (10)	0.0005 (10)
C9	0.0136 (12)	0.0213 (13)	0.0238 (13)	0.0014 (10)	0.0021 (10)	0.0009 (10)
C10	0.0152 (13)	0.0407 (17)	0.0281 (15)	0.0001 (11)	−0.0034 (11)	−0.0030 (12)
C11	0.0188 (13)	0.0265 (14)	0.0193 (13)	0.0039 (10)	0.0034 (10)	0.0032 (10)
C12	0.0288 (14)	0.0318 (15)	0.0181 (13)	0.0018 (12)	0.0038 (11)	0.0003 (11)
C13	0.0357 (17)	0.0392 (18)	0.0371 (17)	−0.0026 (14)	0.0202 (13)	−0.0049 (14)
C14	0.0206 (14)	0.0311 (16)	0.0436 (17)	−0.0031 (12)	0.0088 (12)	0.0001 (13)
C15	0.0232 (13)	0.0258 (14)	0.0250 (13)	0.0000 (11)	0.0049 (10)	−0.0001 (11)
O5	0.0157 (9)	0.0397 (12)	0.0191 (9)	0.0004 (8)	0.0029 (7)	0.0009 (8)
O6	0.0131 (8)	0.0313 (10)	0.0158 (9)	−0.0018 (7)	0.0012 (6)	−0.0015 (7)
O7	0.0166 (8)	0.0252 (10)	0.0177 (9)	−0.0024 (7)	−0.0007 (7)	−0.0008 (7)
O8	0.0128 (9)	0.0396 (12)	0.0231 (10)	−0.0041 (8)	0.0028 (7)	−0.0010 (8)
N2	0.0145 (10)	0.0433 (15)	0.0186 (11)	−0.0029 (10)	−0.0007 (8)	−0.0006 (10)
C16	0.0140 (12)	0.0244 (14)	0.0220 (13)	0.0031 (10)	0.0026 (10)	0.0007 (10)
C17	0.0123 (12)	0.0291 (15)	0.0238 (13)	−0.0007 (10)	−0.0006 (10)	−0.0001 (11)
C18	0.0179 (13)	0.0223 (13)	0.0199 (13)	0.0016 (10)	−0.0009 (10)	0.0006 (10)
C19	0.0173 (13)	0.0201 (13)	0.0203 (13)	−0.0005 (10)	0.0001 (10)	0.0005 (10)
C20	0.0174 (12)	0.0204 (13)	0.0173 (12)	0.0028 (10)	0.0029 (9)	0.0010 (10)
C21	0.0172 (12)	0.0219 (13)	0.0174 (12)	0.0012 (10)	−0.0012 (9)	0.0001 (10)
C22	0.0197 (13)	0.0295 (15)	0.0163 (12)	−0.0008 (11)	0.0000 (10)	−0.0010 (10)
C23	0.0194 (13)	0.0294 (15)	0.0184 (13)	−0.0003 (11)	0.0055 (10)	0.0013 (10)
C24	0.0160 (12)	0.0215 (13)	0.0247 (13)	−0.0017 (10)	0.0010 (10)	0.0013 (10)
C25	0.0157 (12)	0.0407 (17)	0.0289 (15)	−0.0029 (12)	0.0073 (10)	0.0013 (12)
C26	0.0179 (13)	0.0349 (16)	0.0196 (13)	−0.0031 (11)	−0.0010 (10)	0.0068 (11)
C27	0.0278 (15)	0.054 (2)	0.0178 (13)	0.0073 (14)	−0.0013 (11)	−0.0015 (13)
C28	0.0281 (15)	0.0321 (16)	0.0352 (16)	0.0000 (12)	−0.0121 (12)	−0.0022 (12)
C29	0.0224 (14)	0.0341 (17)	0.0409 (17)	0.0075 (12)	−0.0003 (12)	0.0044 (13)
C30	0.0345 (15)	0.0259 (15)	0.0266 (14)	0.0012 (12)	−0.0040 (11)	0.0003 (11)
O1W	0.0633 (15)	0.0337 (12)	0.0203 (10)	−0.0172 (11)	−0.0063 (10)	0.0017 (9)
O2W	0.0448 (12)	0.0328 (12)	0.0201 (10)	0.0148 (10)	0.0079 (8)	0.0028 (8)

### Geometric parameters (Å, º)

**Table:**

O1—C1	1.233 (3)	O6—C16	1.390 (3)
O2—C5	1.374 (3)	O7—C21	1.372 (3)
O2—C1	1.391 (3)	O7—C26	1.468 (3)
O3—C6	1.374 (3)	O8—C24	1.358 (3)
O3—C11	1.465 (3)	O8—C25	1.430 (3)
O4—C9	1.358 (3)	N2—C18	1.331 (3)
O4—C10	1.431 (3)	N2—H2A	0.8800
N1—C3	1.329 (3)	N2—H2B	0.8800
N1—H1A	0.8800	C16—C17	1.399 (4)
N1—H1B	0.8800	C17—C18	1.381 (4)
C1—C2	1.398 (4)	C17—H17	0.9500
C2—C3	1.384 (4)	C18—C19	1.462 (3)
C2—H2	0.9500	C19—C20	1.395 (4)
C3—C4	1.462 (3)	C19—C22	1.396 (4)
C4—C5	1.391 (3)	C20—C21	1.398 (4)
C4—C7	1.399 (4)	C21—C24	1.397 (4)
C5—C6	1.397 (3)	C22—C23	1.385 (4)
C6—C9	1.395 (4)	C22—H22	0.9500
C7—C8	1.378 (4)	C23—C24	1.398 (4)
C7—H7	0.9500	C23—H23	0.9500
C8—C9	1.397 (4)	C25—H25A	0.9800
C8—H8	0.9500	C25—H25B	0.9800
C10—H10A	0.9800	C25—H25C	0.9800
C10—H10B	0.9800	C26—C30	1.510 (4)
C10—H10C	0.9800	C26—C27	1.537 (4)
C11—C15	1.520 (4)	C26—H26	1.0000
C11—C12	1.534 (4)	C27—C28	1.534 (4)
C11—H11	1.0000	C27—H27A	0.9900
C12—C13	1.541 (4)	C27—H27B	0.9900
C12—H12A	0.9900	C28—C29	1.519 (4)
C12—H12B	0.9900	C28—H28A	0.9900
C13—C14	1.524 (4)	C28—H28B	0.9900
C13—H13A	0.9900	C29—C30	1.528 (4)
C13—H13B	0.9900	C29—H29A	0.9900
C14—C15	1.526 (4)	C29—H29B	0.9900
C14—H14A	0.9900	C30—H30A	0.9900
C14—H14B	0.9900	C30—H30B	0.9900
C15—H15A	0.9900	O1W—H1AA	0.8400
C15—H15B	0.9900	O1W—H1AB	0.8400
O5—C16	1.230 (3)	O2W—H2AA	0.8400
O6—C20	1.372 (3)	O2W—H2AB	0.8400
			
C5—O2—C1	120.30 (19)	C21—O7—C26	116.2 (2)
C6—O3—C11	114.45 (19)	C24—O8—C25	118.1 (2)
C9—O4—C10	118.1 (2)	C18—N2—H2A	120.0
C3—N1—H1A	120.0	C18—N2—H2B	120.0
C3—N1—H1B	120.0	H2A—N2—H2B	120.0
H1A—N1—H1B	120.0	O5—C16—O6	113.6 (2)
O1—C1—O2	113.8 (2)	O5—C16—C17	127.9 (2)
O1—C1—C2	127.7 (2)	O6—C16—C17	118.4 (2)
O2—C1—C2	118.5 (2)	C18—C17—C16	122.9 (2)
C3—C2—C1	123.0 (2)	C18—C17—H17	118.5
C3—C2—H2	118.5	C16—C17—H17	118.5
C1—C2—H2	118.5	N2—C18—C17	122.0 (2)
N1—C3—C2	121.8 (2)	N2—C18—C19	120.2 (2)
N1—C3—C4	120.5 (2)	C17—C18—C19	117.8 (2)
C2—C3—C4	117.7 (2)	C20—C19—C22	118.0 (2)
C5—C4—C7	117.8 (2)	C20—C19—C18	117.6 (2)
C5—C4—C3	117.6 (2)	C22—C19—C18	124.4 (2)
C7—C4—C3	124.5 (2)	O6—C20—C19	122.5 (2)
O2—C5—C4	122.8 (2)	O6—C20—C21	115.3 (2)
O2—C5—C6	115.1 (2)	C19—C20—C21	122.2 (2)
C4—C5—C6	122.1 (2)	O7—C21—C24	119.8 (2)
O3—C6—C9	120.6 (2)	O7—C21—C20	121.8 (2)
O3—C6—C5	120.5 (2)	C24—C21—C20	118.3 (2)
C9—C6—C5	118.7 (2)	C23—C22—C19	121.0 (2)
C8—C7—C4	121.3 (2)	C23—C22—H22	119.5
C8—C7—H7	119.4	C19—C22—H22	119.5
C4—C7—H7	119.4	C22—C23—C24	120.1 (2)
C7—C8—C9	120.1 (2)	C22—C23—H23	120.0
C7—C8—H8	119.9	C24—C23—H23	120.0
C9—C8—H8	119.9	O8—C24—C21	114.7 (2)
O4—C9—C6	115.0 (2)	O8—C24—C23	124.9 (2)
O4—C9—C8	125.0 (2)	C21—C24—C23	120.4 (2)
C6—C9—C8	120.0 (2)	O8—C25—H25A	109.5
O4—C10—H10A	109.5	O8—C25—H25B	109.5
O4—C10—H10B	109.5	H25A—C25—H25B	109.5
H10A—C10—H10B	109.5	O8—C25—H25C	109.5
O4—C10—H10C	109.5	H25A—C25—H25C	109.5
H10A—C10—H10C	109.5	H25B—C25—H25C	109.5
H10B—C10—H10C	109.5	O7—C26—C30	111.6 (2)
O3—C11—C15	111.2 (2)	O7—C26—C27	106.3 (2)
O3—C11—C12	106.6 (2)	C30—C26—C27	105.4 (2)
C15—C11—C12	105.6 (2)	O7—C26—H26	111.1
O3—C11—H11	111.1	C30—C26—H26	111.1
C15—C11—H11	111.1	C27—C26—H26	111.1
C12—C11—H11	111.1	C28—C27—C26	106.3 (2)
C11—C12—C13	106.2 (2)	C28—C27—H27A	110.5
C11—C12—H12A	110.5	C26—C27—H27A	110.5
C13—C12—H12A	110.5	C28—C27—H27B	110.5
C11—C12—H12B	110.5	C26—C27—H27B	110.5
C13—C12—H12B	110.5	H27A—C27—H27B	108.7
H12A—C12—H12B	108.7	C29—C28—C27	104.6 (2)
C14—C13—C12	105.2 (2)	C29—C28—H28A	110.8
C14—C13—H13A	110.7	C27—C28—H28A	110.8
C12—C13—H13A	110.7	C29—C28—H28B	110.8
C14—C13—H13B	110.7	C27—C28—H28B	110.8
C12—C13—H13B	110.7	H28A—C28—H28B	108.9
H13A—C13—H13B	108.8	C28—C29—C30	102.5 (2)
C13—C14—C15	103.0 (2)	C28—C29—H29A	111.3
C13—C14—H14A	111.2	C30—C29—H29A	111.3
C15—C14—H14A	111.2	C28—C29—H29B	111.3
C13—C14—H14B	111.2	C30—C29—H29B	111.3
C15—C14—H14B	111.2	H29A—C29—H29B	109.2
H14A—C14—H14B	109.1	C26—C30—C29	104.1 (2)
C11—C15—C14	103.8 (2)	C26—C30—H30A	110.9
C11—C15—H15A	111.0	C29—C30—H30A	110.9
C14—C15—H15A	111.0	C26—C30—H30B	110.9
C11—C15—H15B	111.0	C29—C30—H30B	110.9
C14—C15—H15B	111.0	H30A—C30—H30B	108.9
H15A—C15—H15B	109.0	H1AA—O1W—H1AB	106.9
C20—O6—C16	120.44 (19)	H2AA—O2W—H2AB	106.9
			
C5—O2—C1—O1	179.3 (2)	C20—O6—C16—O5	175.9 (2)
C5—O2—C1—C2	−0.3 (4)	C20—O6—C16—C17	−5.0 (4)
O1—C1—C2—C3	−179.9 (3)	O5—C16—C17—C18	179.7 (3)
O2—C1—C2—C3	−0.4 (4)	O6—C16—C17—C18	0.8 (4)
C1—C2—C3—N1	−179.3 (3)	C16—C17—C18—N2	−176.7 (3)
C1—C2—C3—C4	0.6 (4)	C16—C17—C18—C19	4.2 (4)
N1—C3—C4—C5	179.8 (2)	N2—C18—C19—C20	175.8 (3)
C2—C3—C4—C5	−0.2 (4)	C17—C18—C19—C20	−5.2 (4)
N1—C3—C4—C7	−0.1 (4)	N2—C18—C19—C22	−4.9 (4)
C2—C3—C4—C7	179.9 (3)	C17—C18—C19—C22	174.1 (3)
C1—O2—C5—C4	0.7 (4)	C16—O6—C20—C19	4.0 (4)
C1—O2—C5—C6	−179.8 (2)	C16—O6—C20—C21	−176.3 (2)
C7—C4—C5—O2	179.5 (2)	C22—C19—C20—O6	−178.2 (2)
C3—C4—C5—O2	−0.5 (4)	C18—C19—C20—O6	1.2 (4)
C7—C4—C5—C6	0.0 (4)	C22—C19—C20—C21	2.2 (4)
C3—C4—C5—C6	−179.9 (2)	C18—C19—C20—C21	−178.4 (2)
C11—O3—C6—C9	−105.6 (3)	C26—O7—C21—C24	−110.7 (3)
C11—O3—C6—C5	78.9 (3)	C26—O7—C21—C20	73.9 (3)
O2—C5—C6—O3	−2.8 (3)	O6—C20—C21—O7	−6.9 (4)
C4—C5—C6—O3	176.7 (2)	C19—C20—C21—O7	172.7 (2)
O2—C5—C6—C9	−178.4 (2)	O6—C20—C21—C24	177.5 (2)
C4—C5—C6—C9	1.1 (4)	C19—C20—C21—C24	−2.8 (4)
C5—C4—C7—C8	−0.5 (4)	C20—C19—C22—C23	−0.2 (4)
C3—C4—C7—C8	179.4 (3)	C18—C19—C22—C23	−179.5 (3)
C4—C7—C8—C9	−0.1 (4)	C19—C22—C23—C24	−1.1 (4)
C10—O4—C9—C6	175.8 (2)	C25—O8—C24—C21	175.7 (2)
C10—O4—C9—C8	−5.2 (4)	C25—O8—C24—C23	−5.1 (4)
O3—C6—C9—O4	1.8 (4)	O7—C21—C24—O8	5.1 (4)
C5—C6—C9—O4	177.5 (2)	C20—C21—C24—O8	−179.3 (2)
O3—C6—C9—C8	−177.3 (2)	O7—C21—C24—C23	−174.2 (2)
C5—C6—C9—C8	−1.6 (4)	C20—C21—C24—C23	1.4 (4)
C7—C8—C9—O4	−177.8 (3)	C22—C23—C24—O8	−178.7 (3)
C7—C8—C9—C6	1.2 (4)	C22—C23—C24—C21	0.5 (4)
C6—O3—C11—C15	73.9 (3)	C21—O7—C26—C30	66.9 (3)
C6—O3—C11—C12	−171.6 (2)	C21—O7—C26—C27	−178.7 (2)
O3—C11—C12—C13	−107.8 (2)	O7—C26—C27—C28	−111.5 (3)
C15—C11—C12—C13	10.6 (3)	C30—C26—C27—C28	7.1 (3)
C11—C12—C13—C14	14.6 (3)	C26—C27—C28—C29	18.4 (3)
C12—C13—C14—C15	−34.0 (3)	C27—C28—C29—C30	−36.6 (3)
O3—C11—C15—C14	83.5 (2)	O7—C26—C30—C29	85.1 (3)
C12—C11—C15—C14	−31.7 (3)	C27—C26—C30—C29	−29.9 (3)
C13—C14—C15—C11	40.8 (3)	C28—C29—C30—C26	41.4 (3)

### Hydrogen-bond geometry (Å, º)

**Table:**

*D*—H···*A*	*D*—H	H···*A*	*D*···*A*	*D*—H···*A*
N1—H1*A*···O5^i^	0.88	1.99	2.861 (3)	172
N1—H1*B*···O2*W*^ii^	0.88	2.00	2.816 (3)	153
N2—H2*A*···O1^iii^	0.88	2.03	2.888 (3)	166
N2—H2*B*···O1*W*^i^	0.88	2.08	2.838 (3)	144
O1*W*—H1*AA*···O5^iv^	0.84	1.94	2.769 (3)	170
O2*W*—H2*AB*···O1^v^	0.84	1.94	2.767 (3)	168
O1*W*—H1*AB*···O7	0.84	2.17	2.956 (3)	156
O1*W*—H1*AB*···O8	0.84	2.39	2.994 (3)	130
O2*W*—H2*AA*···O3	0.84	2.00	2.833 (3)	171
C7—H7···O2*W*^ii^	0.95	2.60	3.493 (3)	158
C15—H15*B*···O2*W*^vi^	0.99	2.56	3.309 (4)	133
C11—H11···O2	1.00	2.59	3.091 (3)	111
C15—H15*A*···O4	0.99	2.57	3.267 (3)	128
C26—H26···O6	1.00	2.48	3.035 (3)	115

Symmetry codes: (i) −*x*, −*y*+1, −*z*+1; (ii) −*x*+1, *y*+1/2, −*z*+1/2; (iii) −*x*, *y*−1/2, −*z*+1/2; (iv) −*x*, *y*+1/2, −*z*+3/2; (v) −*x*+1, −*y*+1, −*z*; (vi) *x*, *y*+1, *z*.
